# Optimizing social media influencers for health communication in Africa

**DOI:** 10.1080/16549716.2025.2572009

**Published:** 2025-10-14

**Authors:** Hannah Simba, Eshetu Girma

**Affiliations:** Chronic Diseases Management (CDM) Unit, African Population and Health Research Center, Nairobi, Kenya

**Keywords:** Social media, social media influencers, health communication, Africa

## Abstract

The rise of social media has transformed communication, allowing social media influencers to shape opinions and behaviors with unprecedented reach and engagement. In Africa, where traditional health campaigns often struggle to gain traction, social media influencers have the potential to provide a complementary platform and a unique avenue to engage young and targeted audiences, further enhancing the reach and impact of health information. This paper explores the role of influencers in health communication, highlighting their ability to drive trust, engagement, and behavioral change. Drawing on global and African case studies, it highlights the successes of influencer-led health campaigns, such as those promoting vaccinations and mental health awareness. While influencer-driven messaging has great potential, challenges such as misinformation, commercialization, and credibility concerns must be addressed. By strategically utilizing social media influencers, public health initiatives can enhance health literacy, overcome accessibility barriers, and drive positive health outcomes in Africa.

## Paper Context

- **Added knowledge**: This paper highlights the transformative role of social media influencers in health communication, particularly in Africa, where traditional health campaigns may face outreach challenges.
- **Global health impact for policy and action**: By leveraging influencers’ trust and engagement with their audiences, policymakers can enhance public health messaging, improve health literacy, and drive positive behavior change on the continent.

## Background

Since its inception, the internet has revolutionized how we access and share information [1]. With the advent of social media platforms, the internet has evolved from a static information repository into a dynamic, interactive space where individuals can communicate, share ideas, and influence each other’s behavior in real time with enormous reach and frequency [2,3]. Platforms like Instagram, YouTube, LinkedIn, facebook and TikTok have become vital sources of information, especially for younger audiences in Africa. By 2022, the number of social media users in Africa surpassed 384 million [4]. Young people make up the majority of new adopters, positioning them as both the primary audience and drivers of social media growth in Africa [5]. With younger generations spending more time on social media than on traditional media like TV, radio, or print [5–7], social media influencers are uniquely positioned to reach them directly and with greater intensity.

Social media influencers are defined as ‘individuals who have accrued a sizable and engaged following on one or more social media platforms, and who possess the power to shape attitudes, opinions, and behaviors of their audience through their online content’ [8–13] While traditional health campaigns often struggle to gain traction among audiences, influencers present a complementary and unique opportunity to convey health messages in more relatable and engaging ways. Recent scholarship argues that traditional top-down health communication is no longer sufficient in the digital era, and that health institutions must adapt by adopting the communicative strategies that make influencers effective, such as authenticity, relatability, and immediacy [14].

In Africa, the rapid growth of digital platforms offers a unique opportunity to reach youth populations and positively influence health behaviors [5]. Seizing this opportunity can help bridge gaps in health communication caused by limited resources, difficulties accessing services, and Africa’s diverse languages and cultures.

Social media platforms such as Instagram, with their emphasis on visual content and interactive features, have proven effective for shaping consumer behaviors and facilitating brand engagement [15,16]. Their design features enable influencers to amass large followings, collaborate with brands, and sustain active engagement with their audiences [17]. Increasingly, these platforms play a crucial role in health communication. During the COVID-19 pandemic, for example, social media platforms became a primary source of health information for many individuals [18,19]. In a study on the communication needs of cancer patients and their relatives, 80% of cancer patients reported using social media to connect with peers, with many initiating its use after their diagnosis [20]. Health organizations similarly utilize social media for outreach, while health professionals engage with these platforms for a range of health-related purposes [19,21].

Given the ability of these platforms to shape health literacy, attitudes, social norms, and behaviors, they could be strategically leveraged as conduits for health messaging, cues to action and can play an amplifying role of health [22,23]. By tapping into the trust and relatability that influencers have cultivated with their audiences, health campaigns can effectively reach diverse groups, promoting health information in a more engaging and accessible way. Beyond communication reach, influencers also act as role models, with studies showing that following them on social media enhances health motivation through relatability [24]. Health has become an increasingly visible and relevant topic within the influencer landscape [25–30], as it resonates with everyday concerns and allows influencers to connect around issues of well-being, lifestyle, and health.

This paper explores how social media influencers can enhance health communication, especially in African settings where traditional campaigns often struggle to engage audiences. It also offers recommendations for leveraging influencers effectively in health messaging (Table 1). These recommendations draw from the case studies and challenges discussed, highlighting the importance of strong partnerships, engaging content, and ongoing evaluation to maximize impact.

**Table 1.** Recommendations for effective use of influencers for public health stakeholders, NGOs, health organizations, and governments.

Recommendation Area	Key Actions
Partnership Models	● Build long-term partnerships with influencers. ● Co-design and pre-test content with influencers where possible. ● Involve influencers in sustained advocacy efforts to enhance engagement and impact. ● Empower healthcare providers on social media skills and working together with influencers
Content Strategy	● Create engaging, accurate, and culturally relevant content. ● Use interactive formats like Q&A sessions, challenges, or behind-the-scenes videos to connect with audiences while sharing health information.
Monitoring & Evaluation	● Track social media metrics (e.g. engagement rates), misinformation and disinformation. ● Establishaudience feedback and response mechanisms. ● Assess changes in health-related behaviors to evaluate the effectiveness of influencer-driven campaigns.

## Building on a long-standing tradition of celebrity advocacy

The use of public figures for health messaging and advocacy is not new. For decades, celebrities such as actors, musicians, and athletes have lent their voices to global health causes, raising awareness, advocacy, and resource mobilization for various issues. For instance, Audrey Hepburn, an actress, used her fame to advocate for children’s rights as a UNICEF Goodwill Ambassador [31]. Similarly, Angelina Jolie, an actress and filmmaker, has been a long-time advocate for refugees as a Special Envoy for the United Nations High Commissioner for Refugees (UNHCR) [32]. Bob Marley, a musician and cultural icon, used his global influence to promote social justice and raise awareness of important health issues, including HIV/AIDS prevention and control [33].

Today’s influencers can serve a similar purpose, but with a more direct and interactive approach. Unlike traditional celebrities (famous for TV, film, music, sport), influencers engage with their audiences in real-time through comments, livestreams, and user-generated content on Instagram, YouTube, Facebook, Twitter, or TikTok [5,8,34].

## The potential of influencers in health communication

Influencers often command large and highly engaged followings, many of whom trust their recommendations implicitly. A 2023 report from Kenya and Nigeria surveyed 1,466 (aged 18 to 60) participants and found that 94.87% actively follow social media influencers [34]. The most followed influencers included celebrities such as musicians and actors (23.08%), journalists and media influencers (18.01%), and industry experts such as fashion designers (16.20%). Notably, 46.89% of respondents reported consistently trusting influencer opinions and regularly relying on their recommendations.

Another survey across Ghana, Kenya, Nigeria, and South Africa found Facebook leading in active user engagement (82%), followed by TikTok (60%), Instagram (54%), Twitter (49%), LinkedIn (28%), and Snapchat (25%) [35]. In Ireland, 59% of survey participants followed influencers on social media, with 16% acknowledging that influencers significantly affected their food choices, and 32% stating they were motivated to make healthier food choices as a result [26]. Importantly, this study also raised concerns around trust, credibility, and the potential spread of false or misleading nutritional information, an issue directly relevant to health communication.

Research comparing Instagram-based celebrities with traditional celebrities further illustrates the potential of influencer marketing. A randomized experiment with 104 participants revealed that Instagram influencers were perceived as more trustworthy, fostering a stronger connection with consumers and generating more positive attitudes toward promoted content [36]. While this study focused on luxury brands, the findings underscore the transferable mechanisms of trust and social presence that can be leveraged for health communication.

For public health professionals, influencers provide access to communities that might be hard to reach through traditional communication channels like television or radio or through other educational interventions [37]. A key advantage of influencer-based campaigns is their ability to target specific demographics, based on age, culture, or shared interests [38–40]. This makes health messaging more relatable and tailored to the audience’s needs. AI technology makes the messaging even more targeted and tailored. Influencers excel in presenting information in creative, engaging, and interactive ways. For example, TikTok challenges and videos or short YouTube videos can make complex health information more digestible and memorable, which improves the retention of important messages [28,30,41,42].

Beyond mainstream influencers, a growing number of social media personalities focus specifically on health-related topics, drawing on lived experiences with conditions such as cancer, dementia, or mental health illnesses, either as patients themselves or as caregivers for loved ones [43]. Their authenticity and vulnerability resonate deeply with their audiences, creating a community of followers who seek information, support, and solidarity.

By targeting these personalities, health campaigns can reach engaged niche audiences, with personal experiences that enhance credibility, relatability, and potential for greater impact on awareness and behavior change.

In addition to lived-experience influencers, health expert content creators such as doctors and nurses are increasingly using social media to share evidence-based health information. A recent study of health expert content creators highlighted both the opportunities and challenges of influencer-driven health communication, emphasizing their role in countering misinformation and overly simplified health messages [27].

## Success stories and case studies

Several health campaigns have successfully incorporated influencers. For instance, during the COVID-19 pandemic, influencers were used to promote vaccination drives, resulting in increased awareness and uptake, particularly among youth. A study in India found that social media influencers significantly impacted their followers’ willingness to get vaccinated against COVID-19 [44]. Between 2021 and 2022, a South African COVID-19 vaccine campaign by Project Last Mile shifted from mass media messaging to targeted influencer-led approaches, using radio DJs, social media micro- and nano-influencers, and faith-based leaders to engage communities with low vaccine uptake [37]. The campaign has reached over 15 million people targeting young people and their parents, resulting in increased vaccination uptake [37]. From 2018 to 2019 a large-scale flu vaccine campaign in the US used micro-influencers to reach African American and Hispanic communities, generating over 17 million impressions and 220,000 engagements across two flu seasons, with 94% of public responses being positive and demonstrating the potential of influencer-driven health campaigns to shift social norms and improve vaccine uptake [45]. Mental health awareness [25,29] and fitness [46] initiatives have also benefited from influencer-led campaigns, which saw spikes in engagement and behavior change.

Recent experimental evidence also shows the effectiveness of influencer-driven health messaging: in a US study, influencer-created posts significantly improved knowledge, attitudes, and intentions to vaccinate against HPV, highlighting their potential to reach audiences where mistrust of traditional health systems is high [47].

In African countries, influencers have strong potential to drive health messages, such as sexual health education, cancer screening, and infectious disease awareness, with early campaigns already demonstrating positive impact.

## Challenges and limitations

The advancement of social media platforms gives the power to communicate anything for anyone with or without proper training of the contents of what is conveyed. Social media is a double-edged sword, with its vast, real-time reach powerful enough to drive social movements and even revolutions [28,48,49]. One of the primary concerns with influencers in health messaging is the risk of misinformation, disinformation, infodemic, message inconsistencies, oversimplified and overgeneralized messaging, and limited information filtering ability (social media literacy) of target audiences [27,28,50]. Some influencers, either unknowingly or intentionally, may promote unverified health claims. Ensuring transparency and selecting credible influencers is crucial to maintain trust and promote accurate health messages.

A key challenge is the risk of health messages being seen as mere advertising, which can erode trust; making authenticity, integrity, careful influencer selection, and scientific accuracy essential. The contents of the messages should be pre-tested for scientific accuracy, cultural issues, media mix, complexity, credibility, completeness, and consistency (pathos, ethos, and logos of communication by Aristotle [51]). Health organizations must ensure that influencers’ values align with public health goals and that they have the expertise or reputation to responsibly communicate health issues.

Health organizations, NGOs, governments, and public health stakeholders should aim to explore and invest in influencer partnerships as part of their broader health communication strategies to ensure more inclusive, effective, and innovative health messaging. Health professionals and health communication scholars should be empowered/skilled on social media use and should consider using social media as a platform for sharing evidence-based information, alongside the growing cadre of health experts who are themselves content creators.

## Data Availability

No data was generated in this study.
